# Molecular mechanisms and multi-target therapeutic strategies of diabetic nephropathy: from pathogenesis to precision interventions

**DOI:** 10.3389/fphar.2025.1729941

**Published:** 2025-12-18

**Authors:** Jiahuan Gong, Xingxing Fang, Xinlei Yao, Fei Xue, Guangdong Qi, Bingqian Chen, Hualin Sun

**Affiliations:** 1 Jiangsu Key Laboratory of Tissue Engineering and Neuroregeneration, Key Laboratory of Neuroregeneration of Ministry of Education, Co-innovation Center of Neuroregeneration, Nantong University, Nantong, Jiangsu, China; 2 Department of Nephrology, The Second Affiliated Hospital of Nantong University, Nantong, Jiangsu, China; 3 Department of Endocrinology, Binhai County People’s Hospital, Yancheng, Jiangsu, China; 4 Department of Orthopedics, Changshu Hospital Affiliated to Soochow University, First People’s Hospital of Changshu City, Changshu, Jiangsu, China

**Keywords:** diabetic nephropathy, molecular mechanisms, multi-target therapeutic strategies, precision medicine, signaling pathways

## Abstract

Diabetic nephropathy (DN) has become the primary cause of end-stage renal disease globally, and its epidemiological burden intensifies alongside the surging prevalence of diabetes. The pathogenesis involves complex interactions among metabolic dysregulation, oxidative stress, inflammatory responses, and fibrotic signaling pathways. Hyperglycemia drives renal injury through activation of the RAAS and accumulation of advanced glycation end products (AGEs), while aberrant activation of key signaling pathways such as TGF-β/Smad3 and NF-κB further promotes renal fibrosis. Current clinical diagnosis primarily relies on proteinuria and glomerular filtration rate indicators, yet their insufficient sensitivity for early renal injury leads to high underdiagnosis rates of nonproteinuric DN. Traditional therapy, centered on renin-angiotensin system blockers, can delay disease progression but fails to reverse renal fibrosis. Recent years have witnessed significant therapeutic breakthroughs. These include SGLT2 inhibitors improving glomerular hypertension via mechanisms independent of glucose-lowering, novel anti-inflammatory and anti-fibrotic agents such as nonsteroidal mineralocorticoid receptor antagonists targeting TGF-β/Smad3 pathway inhibition, and multi-target traditional Chinese medicine interventions offering comprehensive protection by regulating signaling networks like PI3K/Akt and AGE-RAGE. At the molecular level, research reveals that inflammation and immune dysregulation, oxidative stress and metabolic disorders, epigenetic modifications, and cellular structural and functional damage collectively form the intricate pathological network of diabetic nephropathy. Emerging technologies like nanodrug delivery systems, stem cell therapy, and gene editing show broad prospects for precise interventions targeting specific molecular pathways. Future research must integrate multi-omics technologies to deeply dissect disease heterogeneity, develop efficient biomarkers for early diagnosis, and optimize therapeutic efficacy through innovative drug delivery systems, while strengthening evidence-based validation of integrated traditional Chinese and Western medicine strategies. This approach will provide novel insights for the precise prevention and control of diabetic nephropathy.

## 1 Introduction

Diabetic nephropathy (DN) has emerged as the primary global cause of ESRD, accounting for over 40% of ESRD case (Wu et al., 2023). In China, DN serves as a major contributor to chronic kidney disease (CKD), with the soaring prevalence of diabetes leading to rising mortality from renal failure and cardiovascular complications (Wu et al., 2023; He et al., 2024a). The global diabetic population is projected to reach 592 million by 2035, imposing a substantial socioeconomic burden due to DN-related healthcare expenditures (Jairoun et al., 2023). Epidemiological studies show that about 30%–40% of diabetic patients develop diabetic nephropathy (DN). Among them, approximately 30% of those with type 1 diabetes and 40% with type 2 diabetes eventually progress to end-stage renal disease (ESRD), necessitating treatment such as dialysis or kidney transplantation (Jairoun et al., 2023; Li et al., 2021). Furthermore, DN patients face significantly elevated cardiovascular event risks and exhibit higher mortality rates compared to non-DN diabetic individuals (Hu et al., 2022a). Collectively, the escalating prevalence of DN and its associated multiple health threats constitute a major global public health challenge.

**FIGURE 1:**
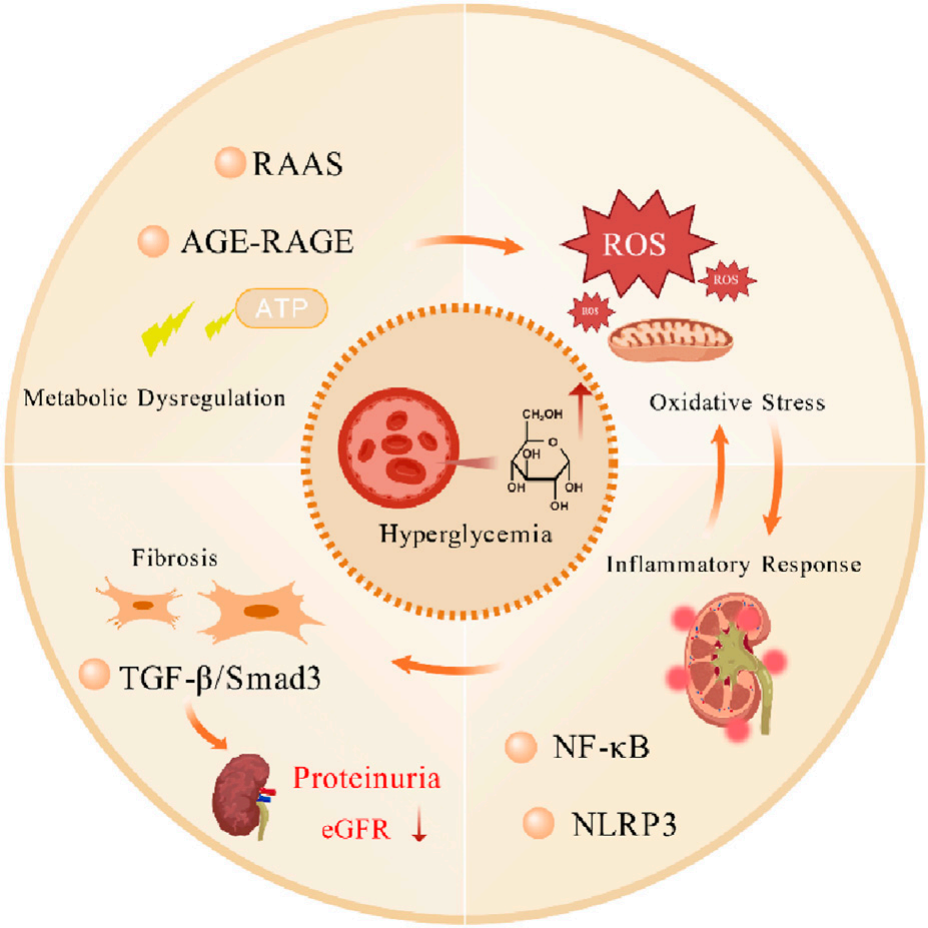
Molecular pathogenic network of diabetic nephropathy. Diabetic nephropathy originates from the persistent elevation of blood glucose levels, which leads to metabolic disorders, manifested as the dysregulation of the RAAS and the AGE-RAGE axis, promoting an increase in intracellular ROS levels and leading to significant oxidative stress. This also triggers an inflammatory response, mediated through the activation of key pro-inflammatory signaling molecules, such as NF-κB and the NLRP3 inflammasome, forming a vicious cycle of oxidative stress and inflammation. Meanwhile, the activation of the TGF-β/Smad3 signaling pathway promotes the occurrence of renal fibrosis, a hallmark of diabetic nephropathy, ultimately presenting as proteinuria and a decline in eGFR, indicating the deterioration of renal function. Created with BioGDP.com.

The pathogenesis of DN involves complex interactions among metabolic dysregulation, oxidative stress, inflammatory responses, and fibrotic signaling pathways (Figure 1). Sustained hyperglycemia activates the RAAS, inducing glomerular hypertension and vascular injury while promoting the accumulation of AGEs. This accumulation drives inflammatory cytokine release, such as TNF-α and IL-6, and oxidative stress through the AGE-RAGE signaling axis (Hudkins et al., 2022; Elendu et al., 2023). Aberrant activation of key molecular pathways, notably TGF-β/Smad3, induces renal tubular epithelial-mesenchymal transition (EMT) and extracellular matrix deposition, thereby accelerating renal fibrosis (He et al., 2024b). Mitochondrial dysfunction also constitutes a core mechanism, as the hyperglycemic environment causes excessive reactive oxygen species (ROS) generation and mitochondrial DNA leakage, which activates macrophages via Toll-like receptor 9 (TLR-9) and amplifies renal inflammation (Ito et al., 2022). Current standard therapies primarily rely on RAAS blockers, such as ACE inhibitors or angiotensin receptor blockers (ARBs), combined with glycemic and blood pressure control; however, these approaches can only delay rather than halt disease progression (Kim et al., 2022). Therefore, a deeper understanding of these multi-target interaction networks is urgently needed to develop breakthrough therapies (Dong et al., 2024). Thus, simultaneous targeting of these interconnected mechanisms may represent a promising strategy for developing more effective interventions against diabetic nephropathy.

Proteinuria, measured by urinary albumin-to-creatinine ratio (UACR), and eGFR remain the gold standards for clinical diagnosis of DN. However, their insufficient sensitivity for early renal injury and the lack of efficient biomarkers contribute to high rates of missed diagnoses (Pillai and Fulmali, 2023). Notably, diagnosing nonproteinuric DN (NP-DN) presents particular difficulty as it does not follow the classic “proteinuria-renal function decline” progression pattern, causing traditional screening methods to often fail (Samsu, 2021). Molecular mechanisms reveal that the core pathological processes of DN involve intricate cross-talk between multiple pathways. Hyperglycemia activates the RAAS, triggering inflammatory cascades that upregulate NF-κB and TGF-β signaling pathways, thereby promoting renal fibrosis. Concurrently, mitochondrial dysfunction leads to ROS accumulation, exacerbating oxidative stress damage (Ito et al., 2022; Fan et al., 2023; Ji et al., 2021). Furthermore, abnormal expression of molecules associated with podocyte EMT and apoptosis, such as Caspase-3/8, accelerates structural destruction of nephrons (Yasuda et al., 2021). Thus, the complexity of DN’s molecular network poses a significant challenge to early and precise diagnosis, creating an urgent need for novel biomarkers to overcome this clinical bottleneck.

Traditional therapies primarily rely on RAAS blockers, such as ACE inhibitors or ARBs, which can slow albuminuria progression but offer limited efficacy in late-stage DN and fail to reverse renal fibrosis (Samsu, 2021). Recent breakthroughs focus on three key areas. Targeting metabolic regulation, SGLT2 inhibitors like empagliflozin reduce glomerular hypertension through mechanisms independent of glucose-lowering, significantly lowering the UACR and improving podocyte injury, with their renoprotective effects validated in both animal models and clinical trials (Hudkins et al., 2022; Maruyama-Sakurai et al., 2024). In anti-inflammatory and anti-fibrotic approaches, novel nonsteroidal mineralocorticoid receptor antagonists such as finerenone inhibit the TGF-β/Smad3 pathway, alleviating renal interstitial inflammation and collagen deposition (He et al., 2022b), while endothelin receptor antagonists like atrasentan demonstrate therapeutic potential by reducing albuminuria (Ahmad et al., 2021). These emerging therapeutic strategies collectively offer promising avenues to address the unmet clinical needs in advanced DN management.

Multi-target traditional Chinese medicine interventions also demonstrate promise, such as Huangkui capsule (containing quercetin and kaempferol as primary active components), which regulates the PI3K/Akt and AGE-RAGE signaling pathways while improving insulin resistance, inhibiting lipid accumulation, and suppressing NF-κB-mediated inflammation (Niu et al., 2022; An et al., 2023). Similarly, the combination of Astragalus membranaceus and Panax notoginseng synergistically alleviates renal fibrosis via the HIF-1α/JAK2/STAT3 axis (Hu et al., 2022b; Zhao et al., 2023). Furthermore, advances in nanodrug delivery systems like kidney-targeting HDL nanodiscs and gene editing technologies are paving new paths for clinical translation of targeted therapeutic strategies against specific molecular pathways in diabetic nephropathy.

## 2 Search strategy and selection criteria

The literature search for this review was conducted using electronic databases, including PubMed, Web of Science, and Google Scholar, for articles published up to October 2025. The search terms included “diabetic nephropathy,” “diabetic kidney disease,” “molecular mechanisms,” “signaling pathways,” “SGLT2 inhibitors,” “GLP-1 receptor agonists,” “ferroptosis,” “epigenetics,” “nanomedicine,” “gut microbiota,” and “traditional Chinese medicine.” Both original research articles and authoritative reviews were considered. The selection was focused on high-impact studies that provided key insights into the pathogenesis, mechanistic pathways, and emerging therapeutic strategies for diabetic nephropathy. The reference list was subsequently refined to include the most relevant and seminal works.

## 3 Molecular mechanisms of diabetic nephropathy

### 3.1 Inflammation and immune regulation imbalance

**FIGURE 2:**
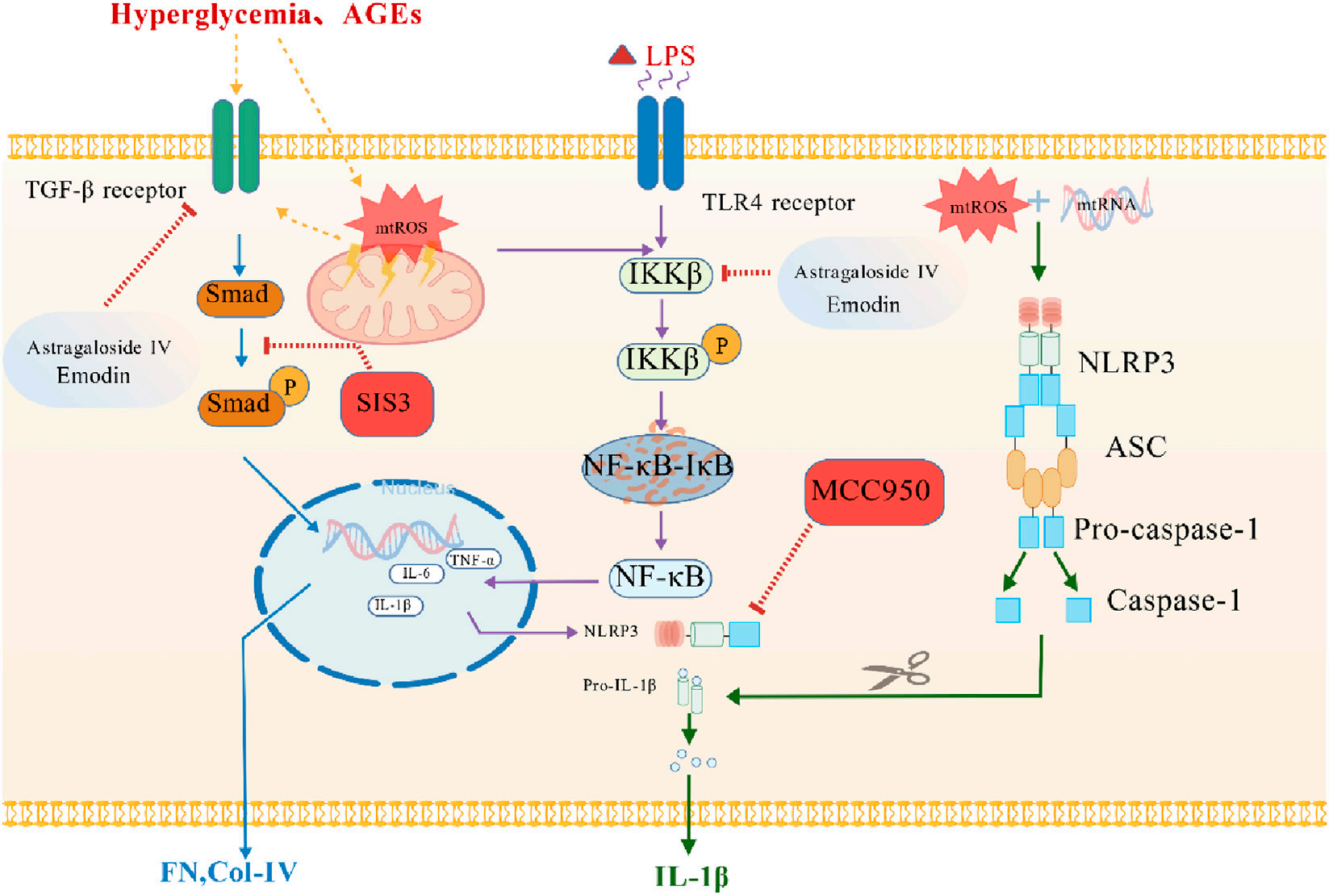
Molecular mechanisms of inflammation and immune dysregulation in diabetic nephropathy. Hyperglycemia and subsequent AGEs activate the Smad pathway through the TGF-β receptor and mtROS. Additionally, LPS acting on the TLR4 receptor activates the IKKβ/NF-κB pathway. They together promote the expression and release of inflammatory factors. The mtROS contribute to the oligomerization of NLRP3 with ASC to form the NLRP3 inflammasome, which cleaves Pro-caspase-1 into its active form, Caspase-1. Active Caspase-1 then processes Pro-IL-1β into the mature, highly inflammatory cytokine IL-1β. These cascades also promote the expression of fibrotic markers like Fibronectin (FN) and Collagen-IV (Col-IV). Astragaloside IV and Emodin are shown to target both the IKKβ/NF-κB and Smad pathways. SIS3, a specific Smad3 inhibitor, blocks the fibrotic signaling. MCC950, a potent and selective NLRP3 inhibitor, directly prevents inflammasome assembly. Created with BioGDP.com.

The progression of DN is closely associated with dysregulated inflammation and immune responses (Figure 2), where the TGF-β/Smad3 signaling pathway serves as a central driver of renal fibrosis. Under persistent hyperglycemia, TGF-β activation promotes Smad3 phosphorylation and nuclear translocation, thereby upregulating extracellular matrix proteins such as fibronectin (FN) and collagen IV (Col-IV). This cascade ultimately leads to mesangial matrix accumulation and tubulointerstitial fibrosis (He et al., 2024b). Studies demonstrate that the Smad3-specific inhibitor SIS3 significantly reduces the urine protein-to-creatinine ratio in db/db mouse models. It also ameliorates glomerulosclerosis and suppresses NF-κB-mediated inflammatory responses, thus confirming the therapeutic targeting value of this pathway (He et al., 2024b; Huang et al., 2024). Furthermore, TGF-β/Smad3 pathway activation correlates with the upregulation of lncRNA Erbb4-IR and LRN9884, which exacerbates renal inflammation and fibrosis (He et al., 2024b). Targeted intervention in the TGF-β/Smad3 signaling cascade therefore not only inhibits fibrosis but also potentially delays DN progression by modulating inflammatory processes.

The progression of DN is closely associated with dysregulated inflammation and immune responses, where the NF-κB-mediated inflammatory cascade serves as a central mechanism. A hyperglycemic environment activates the NF-κB pathway through ROS-dependent IKKβ phosphorylation, promoting its nuclear translocation and upregulating pro-inflammatory factors such as TNF-α, IL-1β, and IL-6 (Pu et al., 2021). These cytokines directly damage podocyte structure and function while recruiting macrophages into the renal interstitium, thereby exacerbating disruption of the glomerular filtration barrier (Burton et al., 2024). Clinical evidence indicates that nuclear localization of the NF-κB p65 subunit in renal tissue correlates significantly with the extent of tubular atrophy and fibrosis in DN patients (D et al., 2023). Furthermore, inflammatory signaling amplifies renal injury by activating the NLRP3 inflammasome, creating a vicious cycle (Ram et al., 2020). Therefore, targeting the NF-κB pathway or its downstream effectors, such as IL-1β, represents a promising strategy to mitigate inflammatory damage in DN. For instance, active components from traditional Chinese medicine, including astragaloside IV and emodin, significantly alleviate renal inflammation and fibrosis by suppressing NF-κB signaling (Wu et al., 2023; Sun et al., 2022). Future research should further explore interactions within the inflammation-immune regulatory network to develop more precise interventions.

In diabetic nephropathy, the NLRP3 inflammasome drives disease progression through a dual-signal activation mechanism. Hyperglycemia significantly upregulates NLRP3 and pro-IL-1β expression via the NF-κB pathway, whereas mitochondrial ROS accumulation and K^+^ efflux trigger inflammasome assembly, activating caspase-1 and promoting IL-1β/IL-18 maturation and release (Ram et al., 2020). Studies reveal a vicious cycle between this pathway and oxidative stress. On one hand, mitochondrial dysfunction induced by high glucose causes ROS bursts that directly activate the NLRP3 inflammasome. On the other hand, IL-1β can feedback-stimulate NADPH oxidase, further exacerbating oxidative stress (Ali et al., 2023). Animal models confirm that NLRP3-knockout db/db mice exhibit reduced proteinuria and significantly attenuated glomerular mesangial matrix expansion (Henstenburg et al., 2024). Preclinical research demonstrates that NLRP3-targeting inhibitors like MCC950 simultaneously suppress inflammasome activation and oxidative stress, lowering renal MDA levels while enhancing SOD activity (He et al., 2022). These findings elucidate the interaction mechanism between inflammation and oxidative stress and provide a crucial basis for developing dual-targeted antioxidant and anti-inflammatory therapies, warranting further exploration of their clinical translation potential.

### 3.2 Oxidative stress and metabolic dysregulation

Mitochondrial dysfunction mediated by the SIRT1/NAD^+^ pathway represents a pivotal mechanism in DN pathogenesis, where oxidative stress emerges as a central driver. SIRT1, an NAD^+^-dependent deacetylase, critically maintains energy metabolism and redox balance. Studies demonstrate that hyperglycemia depletes NAD^+^ levels, consequently inhibiting SIRT1 activity and triggering mitochondrial dysfunction alongside excessive ROS accumulation (Yasuda et al., 2021). This metabolic imbalance further activates inflammatory pathways like NF-κB and fibrotic factors such as TGF-β, accelerating glomerulosclerosis and tubulointerstitial injury (Li et al., 2021). Notably, supplementation with NAD^+^ precursors like nicotinamide mononucleotide (NMN) significantly restores SIRT1 activity, ameliorating proteinuria and renal pathology in db/db mice (Yasuda et al., 2021). Furthermore, natural compounds such as resveratrol activate SIRT1, suppressing p53-mediated apoptosis and enhancing antioxidant defenses (Li et al., 2021). These findings indicate that targeting the SIRT1/NAD^+^ pathway not only alleviates oxidative stress but also improves metabolic disturbances by regulating downstream transcription factors including PGC-1α and FOXO, offering novel therapeutic avenues for DN. Future research should further investigate the crosstalk between this pathway and other metabolic regulators like AMPK and mTOR to optimize intervention strategies.

DN progression is closely linked to dyslipidemia, with aberrant ceramide (Cers) accumulation identified as a key contributor to renal injury (Sun et al., 2022). Study demonstrates that hyperglycemic conditions upregulate Degs2 and Cers transcription, promoting ceramide synthesis and thereby triggering renal lipid deposition and insulin resistance (Sun et al., 2022). Furthermore, ceramide accumulation exacerbates glomerulosclerosis and tubulointerstitial fibrosis by activating oxidative stress and inflammatory responses (Sun et al., 2022). The traditional Chinese medicine Danggui Buxue Decoction (DBT) ameliorates lipid metabolism disorders and renal damage by downregulating Degs2 and Cers expression to reduce ceramide generation while simultaneously promoting Akt phosphorylation (Sun et al., 2022). Similarly, berberine alleviates ceramide-mediated renal fibrosis by modulating iron metabolism and oxidative stress (Wang et al., 2023). These findings suggest that targeting the ceramide metabolic pathway represents a novel therapeutic strategy for mitigating lipotoxic injury in DN. Future research must further elucidate the molecular regulatory networks governing ceramide in DN and its crosstalk with other metabolic pathways.

Ferroptosis represents an iron-dependent form of programmed cell death characterized by excessive intracellular accumulation of lipid peroxides and inhibition of glutathione peroxidase 4 (GPX4) activity (Mao et al., 2023; Wang et al., 2022; Hong et al., 2022). In DN, hyperglycemic conditions trigger ferroptosis through multiple pathways, exacerbating renal cell damage. Studies demonstrate that high glucose levels induce iron accumulation within renal tubular epithelial cells and podocytes, activating NADPH oxidase (NOX) and generating ROS, thereby initiating lipid peroxidation chain reactions (Wei et al., 2023). Furthermore, downregulation of GPX4 expression in the diabetic state leads to the collapse of the antioxidant defense system, further promoting ferroptosis occurrence (Tian et al., 2024; Wang et al., 2024). Key regulators of ferroptosis, such as ACSL4 and TFR1, are significantly upregulated in renal tissues of DN patients, showing positive correlations with the severity of renal fibrosis and proteinuria (Wu et al., 2023). Notably, ferroptosis also activates the NLRP3 inflammasome by releasing damage-associated molecular patterns (DAMPs), creating a detrimental “ferroptosis-inflammation” vicious cycle that accelerates renal function deterioration (Ram et al., 2020; Din et al., 2024). Recently, multiple studies have found that traditional Chinese medicine monomers like baicalein and curcumin can inhibit ferroptosis by modulating the Nrf2/HO-1 and System Xc-/GPX4 pathways, thereby alleviating renal injury in DN models (Zhuang et al., 2024). These discoveries offer new insights into ferroptosis-targeted therapeutic strategies, and future research needs to further explore the translational potential of specific ferroptosis inhibitors for clinical DN treatment.

Furthermore, emerging evidence reveals significant crosstalk between ferroptosis and core DN pathways. Ferroptosis-derived lipid peroxides and DAMPs can activate the TGF-β/Smad3 pathway, creating a feed-forward loop that exacerbates renal fibrosis (Dwivedi and Sikarwar, 2024). Concurrently, the NF-κB pathway, a master regulator of inflammation, can be activated by ferroptotic stress, leading to the upregulation of pro-ferroptotic mediators like NOX, thereby intertwining inflammatory and ferroptosis mechanisms (Hong et al., 2025). This intricate crosstalk underscores the potential of dual-targeting strategies that simultaneously inhibit ferroptosis and key fibrotic/inflammatory pathways.

### 3.3 Mitochondrial quality control in DN

Mitochondrial quality control, including dynamics (fusion/fission) and mitophagy, is essential for maintaining renal cellular integrity (Sun et al., 2024). In DN, hyperglycemia disrupts this balance by upregulating fission proteins such as Drp1 and suppressing fusion proteins like Mfn2, leading to mitochondrial fragmentation and impaired function (Xuan et al., 2025). Under physiological conditions, damaged mitochondria are eliminated through mitophagy, a selective autophagic process. However, mitophagic flux is compromised in DN, resulting in the accumulation of dysfunctional organelles that exacerbate ROS production and promote apoptosis and inflammation. Key regulatory pathways, including PINK1/Parkin and BNIP3/Nix, are also dysregulated in the diabetic kidney. Preclinical studies suggest that restoring mitochondrial quality control—by enhancing mitophagy or curbing excessive fission—holds therapeutic potential for alleviating DN (Mise et al., 2020). Targeting these mechanisms may thus offer a promising avenue for renoprotection in diabetes.

### 3.4 Epigenetics and signaling pathways

Long non-coding RNAs (lncRNAs) play significant roles in diabetic nephropathy (DN) by regulating the TGF-β/Smad3 signaling pathway. Studies indicate that lncRNAs Erbb4-IR and LRN9884 participate in renal fibrosis and inflammation during DN progression by influencing Smad3 activity (He et al., 2024b). In the db/db mouse model, the Smad3 inhibitor SIS3 significantly suppresses TGF-β/Smad3 pathway activation, thereby alleviating renal fibrosis and inflammation while concurrently upregulating Smad7 expression and restoring the balance of the TGF-β/Smad signaling axis (He et al., 2024b). Furthermore, lncRNA MALAT1 exacerbates fibrosis in renal tubular epithelial cells by promoting Smad3 phosphorylation, whereas inhibiting MALAT1 expression markedly improves DN pathological progression (Casagrande et al., 2021). These findings reveal the crucial regulatory function of lncRNAs within the TGF-β/Smad3 pathway, offering novel molecular targets for DN therapy. Further research into the interactions between lncRNAs and the TGF-β/Smad3 pathway will facilitate the development of more effective DN treatment strategies.

Aberrant microRNA (miRNA) expression plays a key role in vascular injury mechanisms within DN. Research indicates significant alterations in the expression profiles of multiple miRNAs in kidney tissues of DN patients and these changes participate in pathological processes including vascular inflammation, oxidative stress, and fibrosis by regulating downstream target genes. For instance, abnormal levels of miR-126 and miR-342 are closely associated with vascular endothelial dysfunction and their downregulation can activate the vascular endothelial growth factor (VEGF) signaling pathway, consequently exacerbating increased glomerular microvascular permeability and proteinuria formation (Xi et al., 2021). Furthermore, miRNAs such as miR-23-3p and miR-206 affect the proliferation and migration of vascular smooth muscle cells by targeting genes like STAT1 and GJA1, thereby promoting renal tubulointerstitial fibrosis (Dai et al., 2022). Notably, certain traditional Chinese medicine components like quercetin can mitigate DN-related vascular injury by modulating the expression of miRNAs including miR-15-5p and miR-16-5p (Dai et al., 2022). These findings provide a theoretical basis for developing miRNA-based targeted therapeutic strategies and future research should further explore the potential of miRNAs as early diagnostic biomarkers and therapeutic targets for DN.

The AGE-RAGE signaling pathway plays a central role in DN pathogenesis by activating oxidative stress and profibrotic processes, which accelerate kidney injury. Under hyperglycemic conditions, AGEs bind to the receptor RAGE, triggering downstream inflammatory pathways including NF-κB and MAPK. This interaction induces massive ROS generation and proinflammatory cytokine release such as TNF-α and IL-6, exacerbating oxidative damage in glomerular and tubular epithelial cells (Pu et al., 2021; Tian et al., 2024). Simultaneously, AGE-RAGE interactions upregulate TGF-β1 and collagen expression including Col4A1 and FN1, promoting extracellular matrix (ECM) deposition and driving renal fibrosis (Liu et al., 2022a; Bhupesh et al., 2025). Studies reveal that herbal components like quercetin and flavonoids alleviate renal inflammation and fibrosis by inhibiting the AGE-RAGE pathway (Niu et al., 2022; Zhuang et al., 2024), while SGLT2 inhibitors such as empagliflozin improve proteinuria and glomerulosclerosis through indirect modulation of this pathway (Hudkins et al., 2022). Additionally, small-molecule RAGE antagonists like the SUCNR1 inhibitor Cpd3 show potential in suppressing NF-κB activation and EMT progression in animal models (Zhang et al., 2024a). These findings indicate that targeting the AGE-RAGE pathway represents a critical therapeutic strategy for DN, where combined antioxidant and antifibrotic approaches may offer comprehensive renoprotection.

### 3.5 Cellular structural and functional impairment

High-glucose conditions induce podocyte injury through multiple molecular mechanisms, representing a key event in DN progression. Research demonstrates that high glucose activates NADPH oxidase within podocytes, triggering excessive ROS production, which subsequently initiates oxidative stress and mitochondrial dysfunction, ultimately leading to podocyte apoptosis and foot process effacement (Yasuda et al., 2021). Furthermore, high glucose upregulates the TGF-β/Smad3 signaling pathway, promoting podocyte EMT and extracellular matrix (ECM) deposition, thereby exacerbating glomerular injury (He et al., 2024b). Notably, high-glucose environments increase claudin-1 expression while decreasing nephrin expression in podocytes, disrupting glomerular filtration barrier integrity (Yasuda et al., 2021). Sodium-glucose cotransporter 2 inhibitors (SGLT2i), such as empagliflozin, have recently demonstrated significant renoprotective effects in DN treatment. Their mechanism relies not only on glucose-lowering effects but also involves inhibiting the mTORC1 pathway to reduce podocyte apoptosis while upregulating Sirt1 and Nampt expression to activate the NAD + salvage pathway, thereby improving podocyte mitochondrial function (Hudkins et al., 2022; Yasuda et al., 2021). Animal studies further confirm that SGLT2i significantly increase podocyte number, ameliorate foot process architecture, and reduce renal oxidative stress marker levels (Hudkins et al., 2022). These findings provide a solid molecular foundation for applying SGLT2i in podocyte protection for DN. Delving into the molecular mechanisms governing podocyte injury and repair will pave the way for developing novel targeted therapeutic strategies.

Dysregulation of autophagic flux, leading to an imbalance with apoptosis, constitutes a key pathogenic mechanism in diabetic nephropathy (DN)-induced renal injury. The role of autophagy, however, is cell-type-specific. In podocytes, constitutive autophagy is essential for organelle homeostasis, and its impairment under high glucose conditions triggers apoptosis and foot process effacement. In mesangial cells, high glucose suppresses autophagy, thereby promoting extracellular matrix (ECM) deposition and proliferation (Cui et al., 2020). In renal tubular epithelial cells, autophagy can be protective by clearing damaged mitochondria, yet its chronic activation may also contribute to cell death under severe metabolic stress. This cell-specific duality renders autophagy a complex but promising therapeutic target.

Interventions such as SGLT2 inhibitors and Sirt1 activators (e.g., NMN) appear to restore protective autophagy in podocytes, whereas rapamycin analogs can alleviate mesangial matrix expansion (Yu et al., 2024a). Study shows that high glucose inhibits autophagy in human mesangial cells (HMCs), as indicated by a decreased LC3-II/LC3-I ratio and accumulation of the selective autophagy substrate p62. This suppression promotes apoptosis and ECM deposition, accelerating DN progression (Fu et al., 2024). Rapamycin activates the autophagy pathway, significantly increasing the LC3-II/LC3-I ratio while reducing p62 levels, thereby mitigating mesangial proliferation and renal fibrosis (Fu et al., 2024). Furthermore, the Sirt1 activator NMN—an NAD^+^-dependent deacetylase enhancer—improves podocyte injury and glomerulosclerosis by upregulating Sirt1 and the NAD^+^ salvage pathway, including Nampt and Nmnat1. Remarkably, even short-term NMN treatment confers sustained protective effects (Yasuda et al., 2021). The mTORC1 inhibitor ESC-HCM-B nanoplatform, combined with a ROS scavenger, synergistically modulates podocyte energy metabolism, reduces mitochondrial oxidative stress and collagen accumulation, and significantly lowers urinary protein levels in DN rats (Fan et al., 2023). Notably, flavonoids from Huangkui capsules (HKC) inhibit podocyte apoptosis via the PI3K/Akt/mTOR pathway while upregulating Acers and Pdk1 to promote Akt phosphorylation, thereby ameliorating lipid metabolism disorders (Sun et al., 2022). These findings provide multi-layered interventional strategies targeting the autophagy-apoptosis imbalance in DN. Future research should further elucidate how the timing of interventions differentially affects the protection of pancreatic β-cells versus the repair of renal damage.

## 4 Therapeutic strategies for diabetic nephropathy

**FIGURE 3:**
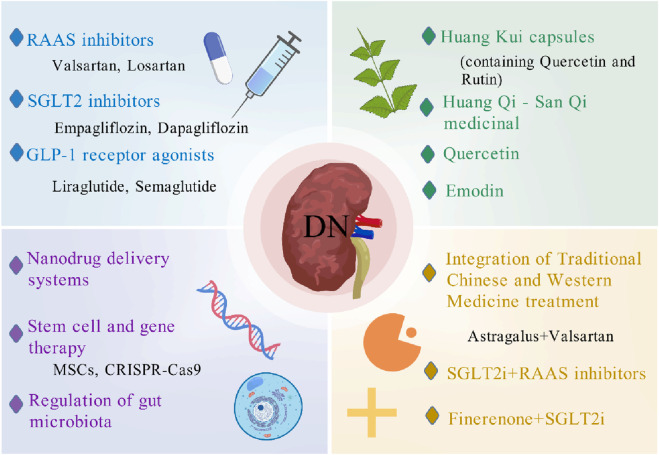
Multi-target therapeutic strategies for diabetic nephropathy. The current and emerging multi-target therapeutic approaches for Diabetic Nephropathy (DN) are categorized as follows: (1) Conventional Western Pharmacotherapies: RAAS Inhibitors (e.g., Valsartan, Losartan); SGLT2 Inhibitors (e.g., Empagliflozin, Dapagliflozin); GLP-1 Receptor Agonists (e.g., Liraglutide, Semaglutide). (2) Natural Products and Traditional Chinese Medicine (TCM) Derivatives: Huang Kui Capsules (containing Quercetin and Rutin); Huang Qi - San Qi medicinal pair; and the purified compounds Quercetin and Emodin. (3) Advanced and Emerging Therapies: Nanodrug Delivery Systems; Stem Cell and Gene Therapy (MSCs and gene-editing tools like CRISPR-Cas9); Regulation of Gut Microbiota as a novel metabolic target. (4) Integrated Treatment Models: Examples include the combination of the TCM herb Astragalus with Valsartan; the concurrent use of SGLT2i and RAAS inhibitors; and the novel Finerenone combined with an SGLT2i. Created with BioGDP.com.

DN requires multifaceted management strategies. This section outlines current and emerging therapeutic approaches, ranging from conventional pharmacotherapy and traditional herbal medicines to advanced nanomedicine and gut microbiota modulation, offering a comprehensive overview of renoprotective interventions (Figure 3) (Table 1).

**TABLE 1:** Key clinical and preclinical studies supporting therapeutic strategies for diabetic nephropathy.

Therapeutic category	Agent/Intervention	Key finding(s)
SGLT2 inhibitors	Empagliflozin	Reduced UACR and improved podocyte injury in BTBR ob/ob mice; renoprotective effects independent of glucose-lowering
SGLT2 inhibitors	Canagliflozin	Significantly lowered UACR and systolic blood pressure in early DN patients
Nonsteroidal MRA	Finerenone	Reduced UACR by up to 40% with lower cardiovascular adverse event rates compared to traditional MRAs
GLP-1 RAs	Liraglutide	Reduced UACR by activating the renal cAMP-PKA pathway and modulating glomerular hemodynamics
Endothelin antagonist	Atrasentan	Demonstrated therapeutic potential by reducing albuminuria in DN patients
TCM formulation	Huangkui capsule	Ameliorated proteinuria in db/db mice via regulating SLC transporters and energy metabolism reprogramming
TCM combination	Astragalus-panax notoginseng	Synergistically alleviated renal fibrosis via the HIF-1α/JAK2/STAT3 axis
Natural compound	Quercetin	Reduced inflammatory factors (TNF-α, IL-1β) and oxidative stress markers by inhibiting PI3K/PKB and TGF-β1/Smad pathways
Natural compound	Isoquercitrin	Mitigated renal inflammation and fibrosis by inhibiting STAT3 phosphorylation and dimerization

^a^ Abbreviations: MRA, mineralocorticoid receptor antagonist; GLP-1, RAs, Glucagon-like peptide-1, receptor agonists; TCM, traditional chinese medicine; UACR, Urine Albumin-to-Creatinine Ratio.

### 4.1 Conventional pharmacotherapy

RAAS inhibitors form the cornerstone of DN treatment by suppressing angiotensin II generation or action, thereby reducing intraglomerular hypertension and proteinuria. Clinical evidence demonstrates that angiotensin-converting enzyme inhibitors (ACEIs) and angiotensin receptor blockers (ARBs) effectively decrease urinary albumin excretion rate (UAER) and slow renal function decline, though their nephroprotective benefits prove limited in advanced DN (Liang et al., 2021). Recently, the novel non-steroidal mineralocorticoid receptor antagonist finerenone exhibits significant advantages, with its high selectivity for renal fibrosis pathways reducing urine albumin-to-creatinine ratio (UACR) by up to 40% while demonstrating lower cardiovascular adverse event rates compared to traditional steroidal antagonists (Hu et al., 2022a). However, hyperkalemia risk persists with these agents and requires close serum potassium monitoring. Therefore, future RAAS-targeted drug development must focus on enhancing therapeutic efficacy while optimizing safety profiles to provide superior treatment options for DN patients.

SGLT2 inhibitors represent a major breakthrough in DN treatment, demonstrating pleiotropic renoprotective mechanisms beyond glucose-lowering effects (Table 2). Research indicates that empagliflozin significantly ameliorates glomerular hyperfiltration while reducing podocyte injury and tubulointerstitial inflammation by inhibiting proximal tubule SGLT2 (Hudkins et al., 2022). At the molecular level, this drug class decreases renal tissue oxidative stress markers and mitigates glomerular basement membrane thickening alongside mesangial matrix expansion (Hudkins et al., 2022). Clinical studies in early DN patients demonstrate that canagliflozin markedly lowers the UACR and systolic blood pressure while also improving the declining trend in eGFR (Zhang and Jiang, 2022). Notably, the cardiorenal protective effects of SGLT2 inhibitors are dose-dependent, with long-term treatment reducing cardiovascular-renal composite endpoint event risk by approximately 30% (Maruyama-Sakurai et al., 2024). Mechanistically, these agents exert protection through multiple pathways including modulation of the tubular hypoxia-inducible factor (HIF) signaling pathway and suppression of sodium-hydrogen exchanger (NHE3) activity (Hudkins et al., 2022). Furthermore, cost-effectiveness analyses reveal superior economic benefits for SGLT2 inhibitors particularly in patients under 70 years old (Maruyama-Sakurai et al., 2024). Collectively, this evidence solidifies the core position of SGLT2 inhibitors in DN management, providing vital therapeutic options for clinical practice. Future research should further explore their synergistic mechanisms with other renoprotective agents.

**TABLE 2:** Comparison between SGLT2 inhibitors and GLP-1 receptor agonists in diabetic nephropathy.

Feature	SGLT2 inhibitors	GLP-1 receptor agonists
Primary mechanism	Inhibit glucose reabsorption in proximal tubules	Activate incretin receptors, enhance insulin secretion
Efficacy in DN	Reduce UACR, slow eGFR decline, cardiorenal protection	Reduce albuminuria, moderate eGFR preservation
Key benefits	Cardio- & renoprotection, weight loss, BP reduction	Glucose control, weight loss, cardiovascular protection
Key risks	Genitourinary infections, euglycemic ketoacidosis	GI side effects (nausea, vomiting), injection-based
Preferred population	T2D with CKD, high cardiovascular risk	T2D with obesity, need for glycemic and weight control

Glucagon-like peptide-1 (GLP-1) receptor agonists demonstrate multi-target synergistic mechanisms in diabetic nephropathy treatment. (Table 2). Research indicates that GLP-1 receptor agonists like liraglutide not only significantly reduce the UACR by activating the renal cAMP-PKA pathway (Yarlagadda et al., 2023) but also directly modulate glomerular hemodynamics and inhibit the TGF-β/Smad3 signaling pathway, thereby attenuating renal fibrosis progression (He et al., 2024b). Their unique cardiorenal protective effects are evidenced by a concurrent reduction in cardiovascular mortality risk (Yarlagadda et al., 2023), which may relate to their suppression of NF-κB-mediated inflammation and improvement of endothelial function. Notably, combining GLP-1 receptor agonists with SGLT2 inhibitors synergistically improves glomerular hyperfiltration and proximal tubule sodium reabsorption through complementary mechanisms (Maruyama-Sakurai et al., 2024), though gastrointestinal adverse events such as nausea and vomiting may affect long-term adherence in approximately 20% of patients (Yarlagadda et al., 2023). Future research should prioritize exploring optimal combination regimens of GLP-1 receptor agonists with the novel mineralocorticoid receptor antagonist finerenone, aiming to develop superior multi-target intervention strategies for diabetic nephropathy.

### 4.2 Chinese herbal medicines and natural bioactive compounds

Chinese herbal formulations synergistically regulate key pathological processes in diabetic nephropathy. Abelmoschus manihot extract, commercially known as Huangkui capsule, specifically modulates SLC transporter activity and significantly ameliorates proteinuria in the db/db mouse model, with mechanisms involving energy metabolism reprogramming in renal tubular epithelial cells (Yu et al., 2023; Yu et al., 2024b). Furthermore, clinical studies demonstrate that Astragalus membranaceus combined with RAAS inhibitors like valsartan significantly enhances overall treatment efficacy, highlighting its role in augmenting conventional therapies (Lin et al., 2024). Additionally, Cordyceps preparations such as Bailing capsule effectively reduce serum creatinine (Scr) and blood urea nitrogen (BUN) levels in patients by modulating Th17/Treg immune balance, thereby slowing renal function deterioration (Sheng et al., 2020). Collectively, these formulations exemplify the therapeutic advantage of a “multi-component, multi-target” approach, particularly suitable for DN as a complex disease involving intertwined pathways. Therefore, evidence-based optimization of herbal formulations represents a promising new direction for clinical practice.

Natural bioactive compounds demonstrate multi-target regulatory advantages in diabetic nephropathy treatment, primarily involving anti-inflammatory, antioxidant, and metabolic modulation mechanisms. Quercetin significantly reduces inflammatory factors (TNF-α, IL-1β) and oxidative stress markers (MDA) while upregulating antioxidant enzymes (SOD, GSH) in DN models by inhibiting PI3K/PKB, AMPK-P38 MAPK, and TGF-β1/Smad pathways (Li et al., 2022). Total flavonoids of Abelmoschus manihot (TFA) ameliorate renal tubular glucose metabolic disorders and reduce urinary protein excretion through regulating SLC transporter families such as slc2a2 and slc5a2 (Yu et al., 2023). Emodin and aloe-emodin from rhubarb alleviate renal fibrosis by targeting key proteins including TP53 and CASP3 to suppress AGE-RAGE and PI3K-Akt pathways (Fu et al., 2022). The Astragalus-Panax notoginseng pairing (ARPN) reduces renal inflammation and fibrotic damage by downregulating HIF-1α and JAK2/STAT3 signaling (Hu et al., 2022b). Rhein specifically inhibits renal interstitial fibrosis progression by activating TP53/CASP3-dependent apoptosis pathways to diminish fibroblast activation (Sun et al., 2022). These compounds exhibit synergistic “multi-component, multi-target, multi-pathway” effects, exemplified by berberine improving renal fibrosis markers (MMP2, TGF-β1) through iron metabolism and oxidative stress regulation (Wang et al., 2023). Future research should integrate nanodelivery technologies like HDL-based nanodrug systems to enhance tissue-specific targeting for precise renal cholesterol metabolism control (He et al., 2022). Natural bioactive compounds provide novel perspectives for DN precision intervention spanning molecular mechanisms to clinical translation.

### 4.3 Emerging therapeutic technologies

**FIGURE 4:**
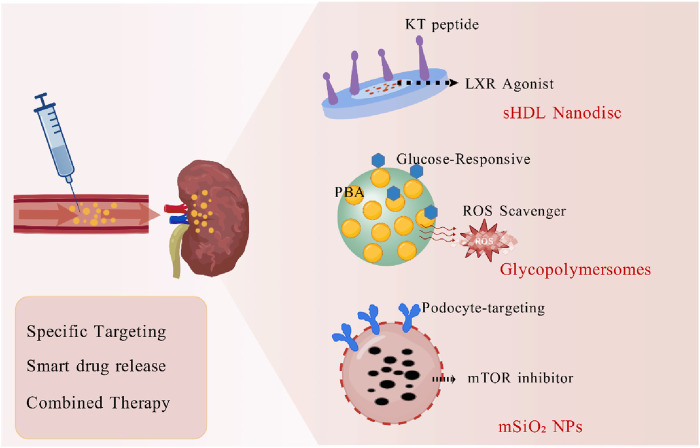
Nanomedicine-Based Strategies for Targeted Therapy in Diabetic Nephropathy. The advanced nanomedicine-based strategies for targeted drug delivery in Diabetic Nephropathy enhance therapeutic efficacy and reduce off-target effects. The sHDL Nanodisc possesses targeted therapy due to KT peptide and LXR Agonist. The glycopolymersomes are engineered to release (Glucose-Responsive) PBA and ROS scavenger. The mTOR inhibitor encapsulated within Podocyte-targeting mSiO2 NPs. These nano-strategies aim to overcome the limitations of conventional drug delivery by improving renal bioavailability, enhancing on-site action, and enabling sophisticated combination regimens for superior treatment of DN. Created with BioGDP.com.

Nanomedicine demonstrates unique advantages for DN treatment, centered on precise renal targeting and synergistic modulation of multiple pathological pathways (Figure 4). Recent research focuses on designing multifunctional nanocarrier platforms. For instance, mTORC1-inhibiting nanoplatforms (ESC-HCM-B) constructed from mesoporous silica nanoparticles (MSNPs) specifically target podocytes while enabling sustained drug release; while the incorporated quantum dots amplify “nanoenzyme” effects, significantly reducing renal ROS levels and improving mitochondrial energy metabolism, thereby delaying glomerulosclerosis (Fan et al., 2023). Furthermore, the rational design of intelligent nanomaterials significantly broadens their application scope. For instance, glycopolymersomes constructed from phenylboronic acid derivative copolymers can dynamically bind blood glucose, enabling sustained regulation of blood glucose levels for up to 32 h. Concurrently, the hydrogen peroxide scavengers encapsulated within these polymersomes effectively alleviate renal oxidative damage, as evidenced by reductions in creatinine and urea levels by 61.7% and 76.6%, respectively (Zhang et al., 2024b). This “drug-free” strategy avoids the metabolic burden of conventional drugs, highlighting nanotechnological innovation. Regarding targeted delivery, synthetic high-density lipoprotein (sHDL) nanodiscs modified with a kidney-targeting ligand (KT peptide) and encapsulating a liver X receptor (LXR) agonist dually regulate lipid metabolism and inflammatory responses in glomerular mesangial cells, significantly reducing urinary protein and suppressing fibrosis (He et al., 2022). Notably, nanocarriers overcome limitations of traditional drugs; polypeptide-based glycopolymersomes synthesized via N-carboxyanhydride ring-opening polymerization ensure biodegradability while maintaining efficacy and safety (Zhang et al., 2024b). These advances underscore how nanomedicine, through spatiotemporal precision, multi-target synergy, and pathological microenvironment responsiveness, provides novel strategies to overcome DN treatment bottlenecks. Future integration of gene editing technologies with nanocarriers, such as kidney-specific delivery of CRISPR-Cas9 systems (Xu et al., 2025), will further propel personalized therapy. The multidimensional intervention strategies of nanomedicine are reshaping the DN therapeutic landscape, offering broad prospects for clinical translation.

Stem cell and gene therapies offer breakthrough strategies for treating DN. Stem cell therapy harnesses multipotent differentiation potential and paracrine effects to repair damaged renal tissue and improve renal function. Mesenchymal stem cells (MSCs) demonstrate significant efficacy in DN animal models due to their anti-inflammatory, anti-fibrotic, and pro-angiogenic properties. For instance, MSCs modulate the immune microenvironment, reducing expression of renal inflammation and fibrosis markers such as TGF-β and α-SMA while promoting repair of glomerular endothelial cells and podocytes (Zhang et al., 2024b). Furthermore, induced pluripotent stem cells (iPSCs) provide avenues for personalized therapy through directed differentiation into renal cells, potentially circumventing immune rejection (Xu et al., 2025). In gene therapy, CRISPR-Cas9 technology enables precise targeting of DN-associated genes. Targeting the TGF-β/Smad3 signaling pathway significantly attenuates renal fibrosis (He et al., 2024b), while adeno-associated virus (AAV)-mediated delivery of the SIRT1 gene activates the NAD + salvage pathway, enhancing renal cell metabolic function (Yasuda et al., 2021). Additionally, combined stem cell-gene approaches, such as MSCs engineered to express ACE2, concurrently regulate the renin-angiotensin system and oxidative stress, achieving synergistic therapeutic effects (Nomura et al., 2021). While challenges persist in cell survival, targeted differentiation efficiency, and vector safety, integrating tissue engineering like 3D bioprinting and nanocarrier technologies broadens clinical translation prospects (Xu et al., 2025). Future research necessitates optimizing stem cell sources and gene editing efficiency to advance these therapies from the laboratory to clinical practice, offering DN patients new hope beyond symptomatic management towards addressing disease etiology.

Gut microbiota modulation represents a promising therapeutic strategy for DN. Studies reveal significant dysbiosis in DN patients characterized by reduced microbial diversity, depletion of beneficial bacteria like short-chain fatty acid (SCFA) producers, and proliferation of opportunistic pathogens. This imbalance exacerbates renal inflammation and fibrosis through the gut-kidney axis (Nagase et al., 2022). For instance, hyperglycemia compromises intestinal barrier integrity, facilitating lipopolysaccharide (LPS) translocation. This activates Toll-like receptor 4 (TLR4) and the NLRP3 inflammasome, thereby promoting renal release of inflammatory cytokines including IL-6 and TNF-α (Ram et al., 2020). These metabolites bind to specific receptors such as GPR43 and GPR109A on renal and immune cells. This binding suppresses the NF-κB pathway and activates the Nrf2 antioxidant pathway, thereby alleviating tubular oxidative stress and renal inflammation (Soltani-Fard et al., 2024). The SCFA-receptor interaction represents a direct molecular link in the gut-kidney axis, translating microbial metabolite production into renal protective signaling (Xiang et al., 2025). Furthermore, fecal microbiota transplantation (FMT) restores microbial balance in animal models, reducing proteinuria and serum creatinine levels. However, rigorous donor screening remains essential for clinical safety (Yang et al., 2024). Traditional Chinese medicine formulations such as Astragalus membranaceus-Panax notoginseng (ARPN) regulate gut microbiota metabolites including L-dopa and cortisol. This action downregulates renal S100a8/S100a9 gene expression and ameliorates albuminuria (Yu et al., 2024b). Notably, Abelmoschus manihot capsules (HKC) combined with irbesartan increase Faecalitalea abundance while reducing Desulfovibrio. This modulates the PI3K/Akt and MAPK pathways to delay DN progression (Diao et al., 2023). Future research should integrate multi-omics technologies like metagenomics and metabolomics to develop personalized microbiota-based regimens and optimize DN treatment efficacy. This approach offers novel multitarget strategies spanning metabolic and immune pathways for DN management.

Emerging research suggests that gut microbiota characteristics may differ between DN subtypes, such as classic proteinuric DN and non-proteinuric DN (NP-DN). For instance, NP-DN might be associated with a distinct microbial signature enriched in bacteria linked to vascular dysfunction (Cheng et al., 2024). Furthermore, the response to interventions like probiotics or TCM (e.g., Huangkui capsule) can vary based on an individual’s baseline microbiota composition. This heterogeneity underscores the potential for personalized microbiota-based regimens (Liu et al. 2022b). Future integration of metagenomics with multi-omics data could enable the stratification of DN patients into microbial sub-types, guiding targeted interventions such as specific probiotic cocktails or prebiotic fibers tailored to correct individual dysbiosis patterns.

### 4.4 Combination therapy strategies

Western-Chinese Medicine Combination Therapy Strategies: Integrative Western-Chinese medicine therapy exhibits significant synergistic effects in treating DN by delaying disease progression through multi-target and multi-pathway mechanisms. Multiple clinical studies confirm that combining conventional Western drugs like renin-angiotensin system (RAS) inhibitors (e.g., irbesartan) with Chinese herbs such as Astragalus membranaceus (Huangqi), Panax notoginseng (Sanqi), and Rheum palmatum (Dahuang) significantly improves renal function markers and reduces proteinuria. For instance, a meta-analysis demonstrated that Astragalus combined with RAS inhibitors markedly increased total efficacy rates while reducing urinary protein excretion rate (UPER), serum creatinine (Scr), and blood urea nitrogen (BUN) levels in stage III DN patients (Lin et al., 2024). Active compounds in rhubarb (e.g., rhein, β-sitosterol) mitigate renal inflammation and fibrosis by modulating AGE-RAGE, PI3K/Akt, and NF-κB signaling pathways (Sun et al., 2022; Fu et al., 2022). Danhong injection combined with alprostadil significantly lowered fasting blood glucose, 24-h urinary protein, and inflammatory factors in DN patients (Hao et al., 2024). Traditional Chinese formulations like Zhenwu Decoction (ZWD) enhanced overall efficacy and improved fasting glucose, BUN, and urinary protein levels when used with conventional Western drugs (Lv et al., 2022). This integrative approach addresses limitations of Western monotherapy in anti-inflammation, antioxidant defense, and anti-fibrosis; berberine alleviates renal injury by suppressing iron overload and oxidative stress (Wang et al., 2023), while Huangkui capsules (HKC) improve insulin resistance via gut microbiota and metabolite regulation (Yu et al., 2024b). Notably, combination therapy maintains a safety profile comparable to Western monotherapy (Zheng et al., 2024). Future large-scale multicenter trials should validate long-term efficacy and explore personalized dosing regimens, offering a novel therapeutic approach for comprehensive DN management.

Multi-Target Pathway Synergistic Therapeutic Strategy: Diabetic nephropathy pathogenesis involves complex interactions across multiple pathways, including metabolic dysregulation, hemodynamic abnormalities, inflammatory responses, and fibrosis. Single-target agents often fail to comprehensively intervene in these processes. Consequently, multi-target drug combinations have become pivotal for overcoming efficacy limitations. The SGLT2 inhibitor and RAAS blocker regimen exemplifies this strategy. SGLT2 inhibitors (e.g., empagliflozin) reduce glucose reabsorption in proximal tubules, alleviating hyperglycemia-induced metabolic stress while increasing urinary sodium excretion to lower intraglomerular hypertension. RAAS blockers (e.g., valsartan) inhibit angiotensin II production or action, mitigating glomerular hyperfiltration and podocyte injury (Hudkins et al., 2022; Pillai and Fulmali, 2023). Clinical studies demonstrate that this combination slows eGFR decline by 35% and reduces cardiovascular event risk (Kim et al., 2022). Its core advantage lies in concurrently addressing metabolic dysregulation and hemodynamic abnormalities, establishing dual metabolic-hemodynamic protection. Additionally, novel nonsteroidal mineralocorticoid receptor antagonists (e.g., finerenone) combined with SGLT2 inhibitors exhibit synergistic anti-inflammatory and antifibrotic effects, expanding therapeutic options (Elendu et al., 2023). Notably, traditional Chinese medicine compounds (e.g., Astragalus-Panax notoginseng combination) modulate multiple pathways like AGE-RAGE, PI3K/Akt, and MAPK signaling, showing unique benefits in reducing proteinuria and ameliorating renal fibrosis (Wu et al., 2023; Król-Kulikowska et al., 2024). This precision combination strategy based on pathological pathways enhances treatment efficacy and broadens personalized therapy possibilities. Future research should explore long-term renoprotective effects of different drug combinations, optimal dosing regimens, and molecular mechanisms to refine clinical management. Multi-target synergistic therapy is emerging as a new paradigm for comprehensive diabetic nephropathy intervention.

## 5 Future research perspectives

DN research is rapidly evolving, integrating cutting-edge approaches like multi-omics, gut-kidney axis insights, advanced drug delivery systems, and modernized traditional Chinese medicine. These innovations are unveiling novel mechanisms and paving the way for precision therapies, significantly improving diagnostic and treatment strategies for this complex disease.

### 5.1 Multi-omics and precision medicine

**FIGURE 5:**
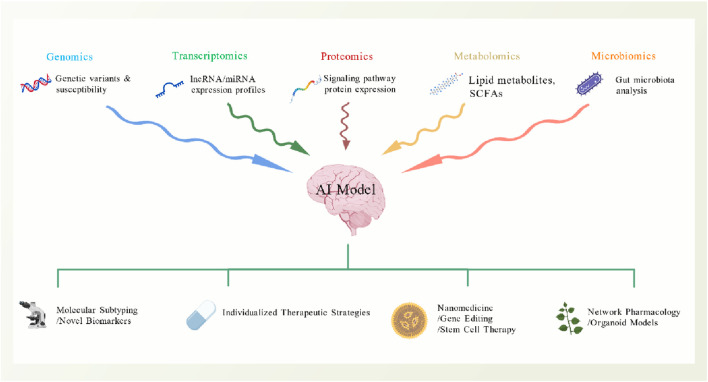
Multi-omics integration and precision medicine framework. The forward-looking framework for understanding and treating Diabetic Nephropathy through the integration of multi-omics data and artificial intelligence, pave the way for precision medicine. Genomics identifies genetic variants and individual susceptibility loci. Transcriptomics analyzes expression profiles, including lncRNA and miRNA. Proteomics identifies the proteins within key signaling pathways. Metabolomics characterizes small-molecule metabolites, such as lipid metabolites and SCFAs. Microbiomics profiles the composition and function of the gut microbiota. These vast, multi-dimensional datasets are then integrated and analyzed by an AI Model. This analysis enables: Molecular subtyping of DN patients and the discovery of novel biomarkers, personalized treatment strategies with advanced modalities (Nanomedicine, Gene Editing technologies and Stem Cell Therapy; Network Pharmacology for multi-target drug discovery; Organoid Models for personalized drug testing). Created with BioGDP.com.

The molecular mechanisms of diabetic nephropathy exhibit considerable complexity and heterogeneity, prompting the application of multi-omics technologies as essential tools for deciphering its pathological underpinnings and developing precise therapeutic strategies (Figure 5). Recent integrative analyses of transcriptomics, proteomics, and metabolomics have unveiled key molecular signatures characteristic of diabetic nephropathy. Analysis of datasets GSE96804 and GSE1009, for instance, identified 729RAAS inhibitor-related targets and 6,039 diabetic nephropathy-associated genes, with CTSC and PDE5A emerging as pivotal targets validated through Mendelian randomization analysis to have a causal relationship with the disease (Zhou et al., 2024). Furthermore, lipidomics investigations revealed that Danggui Buxue Decoction (DBT) ameliorates lipid metabolism dysregulation by downregulating the expression of Degs2 and Cers genes, consequently reducing the accumulation of ceramides (Cers) and phosphatidylethanolamines in the kidney (Sun et al., 2022). Network pharmacology combined with molecular docking techniques has further elucidated the multi-target, multi-pathway mechanisms of action of traditional Chinese medicine (TCM) formulas, such as Zhenwu decoction and the Astragalus membranaceus-Panax notoginseng combination, involving the regulation of signaling pathways including AGE-RAGE, PI3K/Akt, and MAPK (An et al., 2023; Lv et al., 2022). These multi-omics studies not only provide a basis for molecular subtyping of diabetic nephropathy but also lay the groundwork for developing individualized treatment strategies. The application of artificial intelligence and machine learning algorithms enables predictive models based on multi-omics data to more accurately identify high-risk patients and guide personalized therapeutic interventions, marking the transition of diabetic nephropathy diagnosis and treatment into a new era of precision medicine.

### 5.2 Gut-kidney axis regulation

In recent years, the role of the gut–kidney axis in the pathogenesis of DN has garnered increasing attention. Evidence suggests that gut dysbiosis exacerbates DN progression through various mechanisms, including disruption of short-chain fatty acid (SCFA) metabolism, induction of endotoxemia, and activation of systemic inflammation. For example, patients with DN show a decline in beneficial SCFA-producing bacteria such as Faecalibalea and Blautia, alongside an enrichment of pathogenic taxa like Enterobacteriaceae and Desulfovibrio. This microbial imbalance compromises intestinal barrier function, leading to the accumulation of uremic toxins such as indoxyl sulfate. These changes, in turn, activate renal inflammatory pathways—including NF-κB and the NLRP3 inflammasome—and accelerate fibrotic processes (Li et al., 2022; Fu et al., 2022; Zhu et al., 2021). Moreover, gut microbiota-derived metabolites, including D-amino acids and reactive oxygen species, contribute directly to glomerular and tubular dysfunction by promoting oxidative stress and insulin resistance (Nagase et al., 2022). Interventions targeting the gut–kidney axis represent promising therapeutic approaches. Probiotic supplementation, dietary fiber modulation, and fecal microbiota transplantation (FMT) have been shown to restore microbial homeostasis and improve renal outcomes, such as the urinary albumin-to-creatinine ratio and estimated glomerular filtration rate (eGFR) (Wang et al., 2025). However, current research faces several limitations, including high inter-individual variability in gut microbiota composition, reliance on animal models for mechanistic studies, and unresolved issues regarding the long-term safety and standardization of FMT (Gao and Zhang, 2024). Future studies should employ integrated multi-omics strategies—such as metagenomics and metabolomics—to identify specific molecular targets involved in microbiota–host crosstalk. Large-scale clinical trials are also needed to validate personalized microbial interventions (Liu et al., 2025). A deeper understanding of the gut–kidney axis will pave the way for novel precision medicine strategies in DN treatment.

### 5.3 Innovative drug delivery systems

The treatment of DN faces challenges such as poor drug targeting and low bioavailability. Innovative drug delivery systems offer new strategies to address these issues. Nanotechnology demonstrates significant potential in this field. For instance, the multifunctional platform ESC-HCM-B based on mesoporous silica nanoparticles (MSNPs) significantly reduced urinary protein levels and delayed glomerulosclerosis in DN models by targeting the release of mTOR and ROS inhibitors (Fan et al., 2023). Furthermore, sHDL nanodiscs modified with a kidney-targeting ligand (KT peptide) and encapsulating an LXR agonist achieved dual regulation of lipid metabolism and inflammatory responses in glomerular mesangial cells, effectively improving renal function in animal models (He et al., 2022). Glycopolymersomes formed by self-assembly of phenylboronic acid derivative copolymers enabled glucose-responsive drug release and ROS scavenging. Their multi-mechanistic synergistic effects significantly lowered creatinine and urea levels in DN mice (Zhang et al., 2024b). Another study developed cationic polymeric nanocomplexes (mPLM-LSF-OA-CPep) loaded with C-peptide and lisofylline. This system exhibited excellent nephroprotective effects in streptozotocin-induced DN models by extending drug half-life and promoting β-cell regeneration (Singh et al., 2024). These delivery systems offer key advantages including 1) enhanced renal drug accumulation through glomerular filtration or active targeting, 2) intelligent drug release responsive to microenvironmental changes like hyperglycemia or oxidative stress, and 3) integration of multiple therapeutic mechanisms for synergistic efficacy. Future research should further optimize the biocompatibility and scalable manufacturing processes of nanocarriers. Concurrently, exploring the combination of gene editing technologies with delivery systems, such as targeted delivery of CRISPR-Cas9 to kidney-specific cells (Yang et al., 2025), will open new avenues for precision therapeutics in DN.

### 5.4 Modernization research in traditional Chinese medicine

Traditional Chinese medicine (TCM) demonstrates unique multi-target and multi-pathway advantages in DN treatment, yet its modernization faces both challenges and opportunities. Network pharmacology and molecular docking are now widely applied to decipher the mechanisms of TCM formulas, such as Danggui Buxue Decoction (DBT) which ameliorates DN insulin resistance and inflammatory responses by regulating AGE-RAGE, PI3K/Akt, MAPK, and NF-κB signaling pathways (Sun et al., 2022). Rhein and aloe-emodin from rhubarb alleviate renal fibrosis by inhibiting targets like TP53 and CASP3 (Fu et al., 2022). However, the complex composition of TCM and unclear active substance basis present difficulties, as seen with Astragalus membranaceus-Panax notoginseng pairing (ARPN), which significantly reduces urinary protein and improves renal function (An et al., 2023) despite requiring deeper pharmacokinetic studies of its core components. Furthermore, existing clinical research primarily relies on small-sample trials, such as Zhenwu Decoction (ZWD) combined with Western medicine showing superior efficacy over monotherapy (Lv et al., 2022), yet lacking large-scale multicenter validation. Future studies should integrate artificial intelligence and organoid technology, including human pluripotent stem cell (hPSC)-derived kidney organoids to simulate DN pathology (Xu et al., 2025), thereby accelerating TCM target screening and efficacy evaluation. Standardization efforts are equally essential, such as clarifying the metabolic fate of flavonoid components in Huangkui capsules (HKC) via UPLC-MS technology (Diao et al., 2023)and establishing biomarker-based efficacy assessment systems. Ultimately, TCM modernization must overcome the “black box” dilemma by deeply integrating empirical knowledge with contemporary technology to provide precise intervention strategies for DN management.

## 6 Critical analysis of current therapies

While significant progress has been made in DN management, each therapeutic class presents distinct advantages and limitations. SGLT2 inhibitors offer robust cardiorenal protection and metabolic benefits but carry risks of genitourinary infections and euglycemic ketoacidosis. GLP-1 receptor agonists provide multi-target benefits including weight loss and cardiovascular protection, though gastrointestinal side effects limit adherence. Nonsteroidal MRAs like finerenone exhibit potent anti-fibrotic effects with a lower hyperkalemia risk than steroidal agents, yet monitoring remains necessary. RAAS blockers, the long-standing cornerstone, delay but do not halt DN progression and are less effective in advanced stages. Natural products such as Huangkui capsule and quercetin offer multi-target, holistic benefits with minimal side effects, though standardization and large-scale clinical validation are needed. Emerging technologies like nanomedicine and gene editing show precise targeting potential but face challenges in biocompatibility, safety, and clinical translation. A combined approach leveraging the strengths of each modality while mitigating limitations represents the most promising strategy for comprehensive DN management.

## 7 Conclusions and future perspectives

**FIGURE 6:**
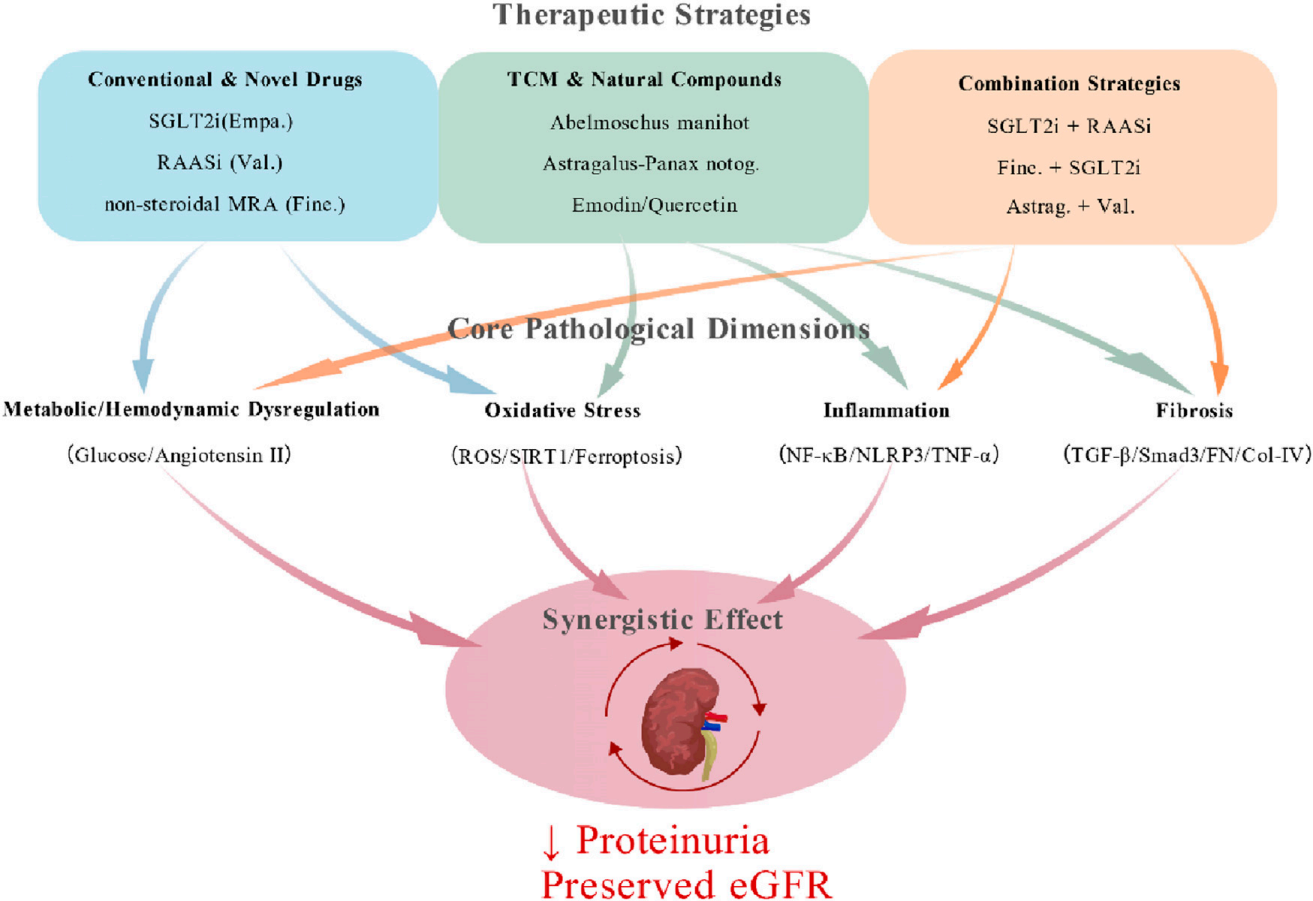
Synergistic multi-target therapy strategies. Conventional & Novel Drugs include SGLT2 inhibitors (e.g., Empagliflozin), RAAS inhibitors (e.g., Valsartan), and novel non-steroidal Mineralocorticoid Receptor Antagonists (MRA, e.g., Finerenone). TCM & Natural Compounds include Abelmoschus manihot, Astragalus-Panax notoginseng herb pair, and active compounds like Emodin and Quercetin. Combination Strategies include SGLT2i + RAASi, Finerenone + SGLT2i, and the integration of Astragalus with Valsartan. These diverse therapeutic agents work in concert to mitigate the key interconnected pathological processes driving DN progression: Metabolic/Hemodynamic Dysregulation (e.g., high Glucose, Angiotensin II); Oxidative Stress (involving ROS/SIRT1/Ferroptosis); Inflammation (NF-κB/NLRP3/TNF-α); Fibrosis (TGF-β/Smad3 pathway, leading to Fibronectin and Collagen-IV deposition). Through the combined therapeutic strategy achieves a superior, synergistic effect, ultimately manifesting in the key clinical hallmarks of renal protection: a significant reduction in proteinuria and the preservation of eGFR. Created with BioGDP.com.

DN is the leading cause of ESRD globally, its pathogenesis involving complex interactions among metabolic dysregulation, oxidative stress, inflammatory responses, and fibrotic pathways (Figure 6). Core molecular mechanisms encompass dysregulated inflammation and immunity, notably abnormal activation of the TGF-β/Smad3 and NF-κB pathways promoting renal fibrosis, while the NLRP3 inflammasome and oxidative stress form a vicious cycle. Metabolic disturbances also play a key role, including SIRT1/NAD^+^ pathway dysfunction causing mitochondrial impairment, ceramide accumulation leading to lipotoxic injury, and ferroptosis exacerbating cellular damage. Epigenetic regulation further contributes via lncRNA/miRNA modulation of critical signaling pathways. Finally, structural cellular damage arises from podocyte EMT and disrupted autophagy-apoptosis balance. Clinical diagnosis primarily relies on proteinuria and eGFR, yet these lack sufficient sensitivity for early nonproteinuric DN (NP-DN). While conventional RAAS inhibitors exhibit limited efficacy, recent therapeutic strategies show significant breakthroughs. Sodium-glucose cotransporter-2 (SGLT2) inhibitors, such as empagliflozin, confer multifaceted renoprotection partly by ameliorating glomerular hypertension. Novel antifibrotic agents like the nonsteroidal mineralocorticoid receptor antagonist (MRA) finerenone target the TGF-β pathway. Traditional Chinese medicine (TCM) formulations and active components, exemplified by Huangkui capsules and quercetin, exert multi-target effects by modulating inflammation, metabolism, and fibrosis pathways. Emerging technologies are opening new avenues for precision intervention, including nanoscale drug delivery systems like renal-targeted sHDL nanodiscs, stem cell and gene therapies employing CRISPR-based editing of the TGF-β pathway, and gut microbiota modulation.

**FIGURE 7:**
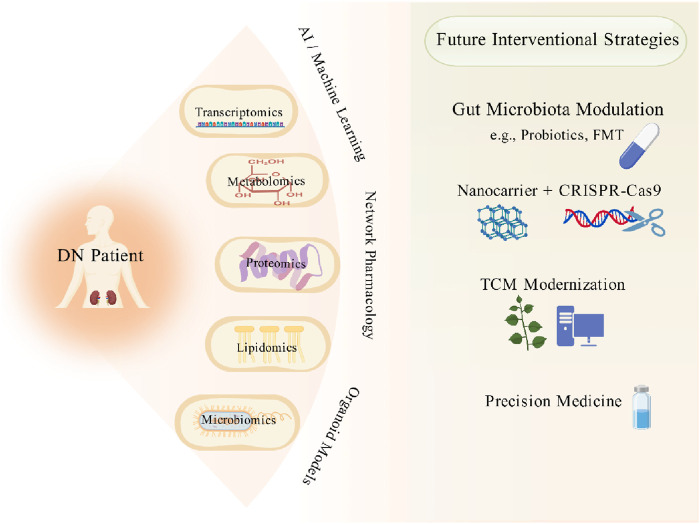
Future research perspectives. The promising future research trajectory for Diabetic Nephropathy centered on the integration of multi-omics data with cutting-edge technologies to achieve precision medicine. The framework is driven by AI and Machine Learning, which serve as the central engine for analyzing complex, multi-layered biological data (Transcriptomics, Metabolomics, Proteomics, Lipidomics, Microbiomics). Network Pharmacology and Organoid Models for elucidating complex drug-target interactions and enabling personalized drug screening. Gut Microbiota Modulation using interventions like Probiotics and Fecal Microbiota Transplantation (FMT) to target the gut-kidney axis. Advanced Biologics and Delivery, such as combining Nanocarriers with gene-editing tools like CRISPR-Cas9 for targeted therapy. TCM Modernization, utilizing the above technologies to validate and optimize Traditional Chinese Medicine formulas. The convergence of these diverse fields ultimately deliver on the promise of Precision Medicine for improved patient stratification and individualized treatment. Created with BioGDP.com.

Future research should focus on four key directions (Figure 7). First, it is essential to integrate multi-omics data—such as transcriptomics and metabolomics—to unravel the heterogeneity of diabetic nephropathy, while employing artificial intelligence to develop early diagnostic biomarkers and personalized treatment strategies. Second, in-depth investigation is needed to elucidate the mechanisms by which microbiota-derived metabolites, such as short-chain fatty acids, influence diabetic nephropathy via immune and oxidative stress pathways, thereby facilitating the clinical translation of probiotics or microbiota transplantation. Third, efforts should be directed toward optimizing nanocarriers like glucose-responsive glycopolymersomes to improve renal targeting and safety, combined with CRISPR technology to achieve precise editing of gene pathways. Additionally, researchers ought to leverage network pharmacology and organoid models to clarify the multi-target mechanisms of traditional Chinese medicine compounds, such as formulations containing Astragali Radix and Panax Notoginseng Radix et Rhizoma, while establishing standardized systems for efficacy evaluation. Through interdisciplinary integration and a combined approach of traditional Chinese and Western medicine, this field may overcome current diagnostic and therapeutic bottlenecks, ultimately shifting the paradigm from delaying disease progression to reversing renal injury.
